# Event-free survival and recurrence patterns after curative resection for locally advanced head and neck squamous cell carcinoma: single-institution real-world outcomes prior to the introduction of perioperative immunotherapy

**DOI:** 10.1007/s10147-026-03092-1

**Published:** 2026-06-16

**Authors:** Takuma Makino, Yuto Naoi, Go Hasegawa, Takuto Yamasaki, Hiroki Noda, Shohei Fujimoto, Junya Matsumoto, Mizuo Ando

**Affiliations:** Department of Otolaryngology - Head and Neck Surgery, Okayama University Graduate School of Medicine, Dentistry and Pharmaceutical Sciences, 2-5-1 Shikata-cho, Kita-ku, Okayama, 700-8558 Japan

**Keywords:** Head and neck squamous cell carcinoma, Surgery, Event-free survival, Recurrence, Salvage surgery

## Abstract

**Background:**

Perioperative immune checkpoint inhibitors have improved outcomes for resectable locally advanced head and neck squamous cell carcinoma (HNSCC). However, real-world surgical outcome data based on contemporary endpoints remain limited.

**Methods:**

We retrospectively analyzed 233 patients who underwent curative-intent surgery for locally advanced HNSCC. Event-free survival (EFS) and overall survival (OS) were evaluated. Unresectable recurrence-free survival (URFS) was explored as an additional endpoint to capture post-recurrence treatment feasibility. Prognostic factors were assessed using Cox proportional hazards models.

**Results:**

The median follow-up duration was 18.2 months. The 1-year EFS and OS rates were 67.1% and 89.4%, respectively. pN2 or higher disease, venous invasion, and perineural invasion were identified as independent adverse prognostic factors. Recurrence occurred in 34.8% of patients; among these, disease control at the last follow-up was achieved in 61.7% following curative-intent salvage treatment. Salvage surgery was associated with significantly improved post-recurrence survival (*p* < 0.001).

**Conclusions:**

This study provides real-world surgical outcome data for resectable locally advanced HNSCC prior to the introduction of perioperative immunotherapy. Recurrence remains a major clinical challenge, with heterogeneous post-recurrence outcomes influenced by treatment feasibility. These findings may help inform interpretation of treatment outcomes and clinical decision-making in the evolving era of perioperative immunotherapy.

**Supplementary Information:**

The online version contains supplementary material available at 10.1007/s10147-026-03092-1.

## Introduction

The management of head and neck squamous cell carcinoma (HNSCC) involves a multidisciplinary approach combining surgery, radiotherapy, and chemotherapy, tailored according to tumor stage and primary site [1–3]. In patients with resectable locally advanced disease, surgery with curative intent remains the central component of treatment, and completion of postoperative adjuvant therapy is critically important, particularly in those with high-risk features [4–6]. However, despite such multimodal treatment, a substantial proportion of patients experience recurrence [7], underscoring the need for further optimization of therapeutic strategies.

In recent years, immune checkpoint inhibitors (ICIs) have demonstrated survival benefits in patients with recurrent or metastatic HNSCC [8], leading to a paradigm shift in treatment. In contrast, conventional perioperative approaches such as neoadjuvant chemotherapy have contributed to organ preservation in some cases but have not yielded clear survival benefits and are considered to have a limited role in HNSCC management [9, 10]. Against this background, the phase III KEYNOTE-689 trial, which evaluated perioperative pembrolizumab in patients with resectable locally advanced HNSCC, demonstrated a significant improvement in event-free survival (EFS) [11, 12], marking a transition to a new era of perioperative treatment strategies.

Treatment outcomes following surgery have traditionally been assessed using endpoints such as overall survival (OS) and disease-free survival. However, these endpoints treat recurrence as a single event and do not adequately capture heterogeneity in post-recurrence clinical courses, particularly with respect to treatment feasibility. Specifically, they do not distinguish between recurrences amenable to salvage surgery and those that are unresectable. Notably, patients who undergo successful salvage surgery after recurrence may achieve long-term survival [13]. These findings suggest that, in addition to recurrence itself, treatment feasibility should be considered a crucial clinical dimension in real-world practice.

Lymphatic invasion, venous invasion, and perineural invasion are well-established pathological features reflecting tumor aggressiveness and the presence of micrometastatic disease, and have been associated with OS [7, 14]. However, their impact not only on recurrence but also on post-recurrence treatment feasibility, particularly the development of unresectable recurrence, has not been sufficiently investigated.

Furthermore, in the real-world setting prior to the introduction of perioperative ICIs, data remain limited for systematic evaluation of outcomes under standard surgical treatment and postoperative adjuvant therapy using EFS definitions aligned with the KEYNOTE-689 trial. Establishing surgery-based real-world reference data may help contextualize the clinical impact of perioperative ICIs.

Based on these considerations, the present study aimed to evaluate EFS and OS in patients with resectable locally advanced HNSCC who underwent curative resection prior to the introduction of perioperative ICIs, using outcome definitions informed by the KEYNOTE-689 trial, and to provide single-institution real-world reference data. In addition, we defined unresectable recurrence-free survival (URFS) as an exploratory endpoint to capture differences in post-recurrence treatment feasibility and investigated associated factors.

## Materials and methods

### Study design and patient population

**Fig. 1:**
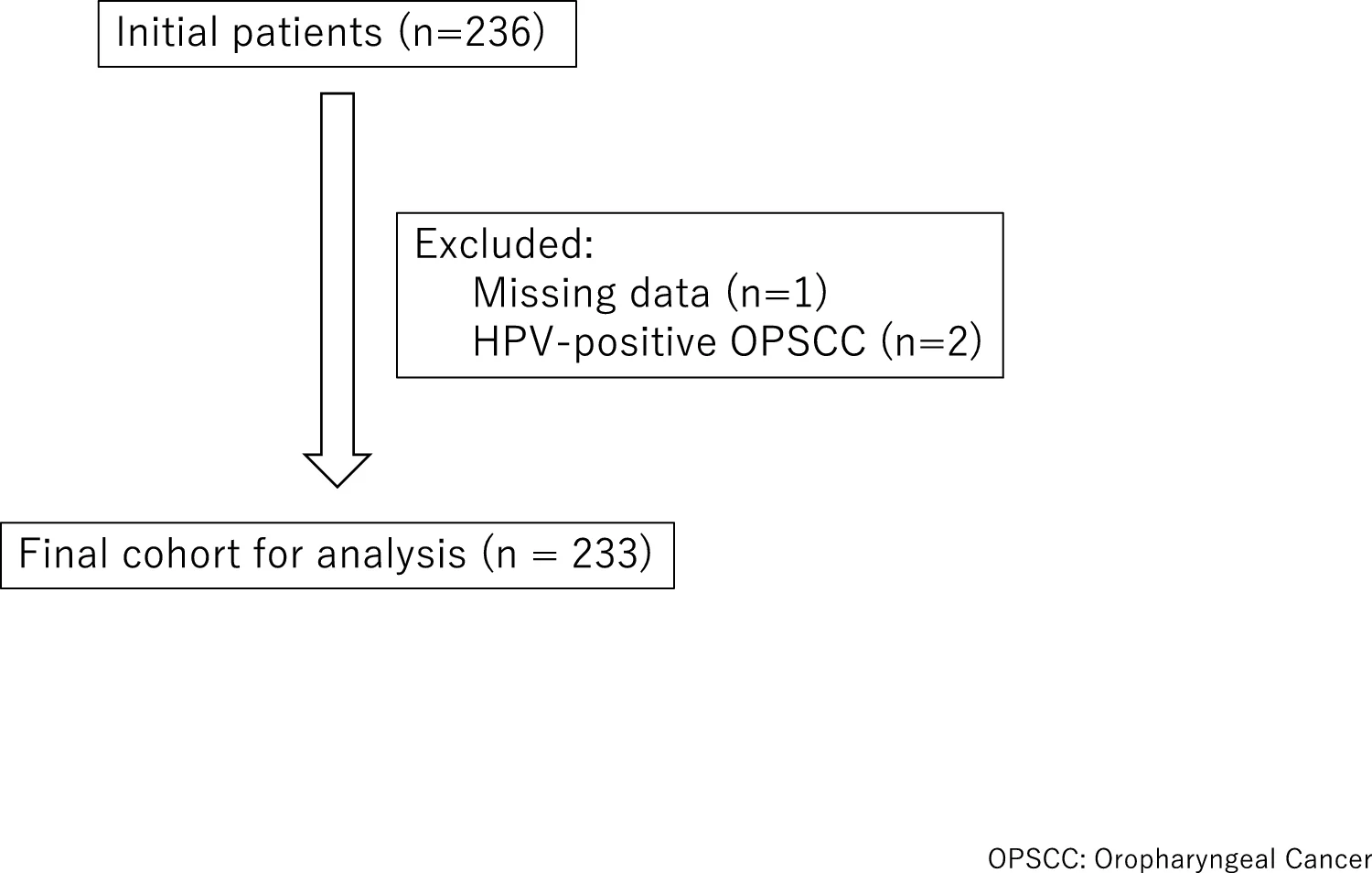
Flowchart of patient selection. A total of 236 patients with resectable locally advanced head and neck squamous cell carcinoma (HNSCC) who underwent curative-intent surgery between January 2020 and December 2025 were initially identified. Patients with incomplete data (n = 1) and those with HPV-positive oropharyngeal cancer (n = 2) were excluded. The final study cohort consisted of 233 patients who met the eligibility criteria

This retrospective study included 236 consecutive patients who underwent surgery as initial treatment for HNSCC at our institution between January 2020 and December 2025 and met eligibility criteria consistent with those of the KEYNOTE-689 trial. After excluding one patient with incomplete data and two with HPV-positive oropharyngeal cancer, 233 patients were included in the final analysis (Fig. 1).

Neoadjuvant chemotherapy was selectively administered primarily in patients expected to experience prolonged waiting time before surgery. Because previous studies have not consistently demonstrated a survival benefit of neoadjuvant chemotherapy in resectable HNSCC, it was not routinely used as a standard strategy for prognosis improvement at our institution. The most commonly used regimens were FP therapy (cisplatin plus 5-fluorouracil) and PCE therapy (carboplatin, paclitaxel, and cetuximab). Pathological risk was categorized based on established adverse features. High-risk disease was defined as the presence of positive surgical margins and/or extranodal extension. Intermediate-risk disease was defined as the absence of high-risk features but the presence of adverse pathological findings, including venous invasion, lymphatic invasion, or perineural invasion.

At our institution, postoperative adjuvant therapy is routinely recommended for patients with high-risk features, typically in the form of concurrent chemoradiotherapy. In contrast, for patients with intermediate-risk features, postoperative radiotherapy was generally not administered during the study period at our institution, although selected patients received adjuvant treatment based on individual clinical considerations such as age, performance status, comorbidities, and patient preference.

To ensure comparability with the KEYNOTE-689 trial, patients were eligible if they met the following criteria: (1) receipt of curative-intent treatment including surgical resection as initial therapy; (2) primary tumor located in the oral cavity, oropharynx, hypopharynx, or larynx; and (3) stage III or IVA disease without distant metastasis (M0) according to the Union for International Cancer Control 8th edition staging system. Exclusion criteria were: (1) p16-positive oropharyngeal cancer; or (2) inability to evaluate primary endpoints due to loss to follow-up. HPV-positive oropharyngeal cancer was excluded to ensure a more homogeneous population, as these tumors represent a biologically distinct entity with a more favorable prognosis and are staged using a separate system incorporating HPV status [15, 16].

### Data collection

Clinical data were retrospectively collected from medical records. Patient-related variables included age, sex, and performance status (PS; ECOG). Tumor-related variables included primary site, clinical T classification, clinical N classification, pathological T classification, pathological N classification, and the presence of extranodal extension. Treatment-related variables included neoadjuvant chemotherapy and postoperative adjuvant therapy. Pathological variables included venous invasion, lymphatic invasion, perineural invasion, and surgical margin status.

### Endpoints and definitions

The primary objective of this study was to evaluate surgical outcomes in a real-world setting prior to the introduction of perioperative immunotherapy, using a framework aligned with the KEYNOTE-689 trial, and to provide historical real-world reference data.

The primary endpoint was EFS, defined with reference to the KEYNOTE-689 trial as the time from the date of surgery to the first occurrence of recurrence (local, regional, or distant) or death from any cause.

The secondary endpoint was OS, defined as the time from the date of surgery to death from any cause. In addition, unresectable recurrence-free survival (URFS) was evaluated as an exploratory endpoint to descriptively capture the clinical heterogeneity of post-recurrence disease, particularly with regard to salvageability. Unresectable recurrence was defined as recurrence not amenable to curative-intent resection at the time of recurrence. This included cases meeting any of the following criteria: (1) technically unresectable disease due to factors such as carotid artery invasion, skull base involvement, or prevertebral fascia invasion; (2) multiple or extensive distant metastases; or (3) medical inoperability due to poor general condition or severe comorbidities. Unresectability was determined at a multidisciplinary tumor board based on anatomical and clinical criteria. Patients who achieved complete control after salvage treatment were not considered to have experienced a URFS event unless subsequent unresectable recurrence or death occurred.

Furthermore, post-recurrence survival (PRS) was defined among patients with recurrence as the time from the date of first recurrence to death from any cause.

### Assessment of recurrence and post-recurrence treatment

Recurrence sites were classified as local, regional, or distant. Simultaneous recurrences occurring across different categories were defined as combined recurrence, whereas multiple lesions within the same category were classified according to that category. Post-recurrence treatment was evaluated with a focus on salvage surgery. Salvage surgery was defined as surgical resection performed with curative intent for recurrent disease. Disease control was defined as the absence of detectable disease at the last follow-up and does not necessarily indicate durable remission. Subsequent recurrence after salvage treatment was evaluated according to the same URFS criteria.

Assessment of resectability and determination of post-recurrence treatment strategies were performed by a multidisciplinary tumor board comprising head-and-neck surgeons, medical oncologists, radiologists, and pathologists. To ensure consistency in evaluation, a retrospective review was conducted by at least two board-certified head-and-neck surgeons. In cases of disagreement, the final decision was reached by consensus.

### Statistical analysis

Continuous variables are summarized as median and range, and categorical variables as frequency and percentage. Survival curves were estimated using the Kaplan–Meier method based on exact date-level intervals, and survival rates at 1 and 3 years were calculated. Cox proportional hazards analyses were also performed using exact date-level intervals. For graphical presentation, time was displayed in months on the x-axis. Differences between groups were compared using the log-rank test.

Prognostic factors for EFS and URFS were analyzed using Cox proportional hazards regression models. Univariable analyses were initially performed, and variables with *p* < 0.10, together with clinically relevant variables selected a priori based on biological plausibility and existing literature (including pN classification, pathological invasion factors, and PS), were considered for inclusion in multivariable models. To avoid overfitting, the number of variables included in multivariable models was restricted according to the number of observed events, maintaining an events-per-variable ratio of approximately ≥ 10. Multicollinearity among candidate variables was assessed using correlation matrices and variance inflation factors (VIF). Variables with a correlation coefficient |r|≥ 0.7 or a VIF > 5 were considered highly correlated and were not included simultaneously in the same model. Age was analyzed as a continuous variable. Venous invasion, lymphatic invasion, and perineural invasion were assessed according to the Japanese Classification of Head and Neck Cancer (grades 0–3) and analyzed as ordinal variables to reflect increasing degrees of invasion severity. A sensitivity analysis was conducted by substituting lymphatic invasion for venous invasion to confirm the robustness of the findings.

Hazard ratios and 95% confidence intervals were calculated. The proportional hazards assumption was evaluated using Schoenfeld residuals and was not violated. Two-sided* p*-values < 0.05 were considered statistically significant. Variable selection differed between models according to endpoint-specific clinical relevance and the number of observed events.

All statistical analyses were performed using IBM SPSS Statistics for Windows (version 26.0; IBM Corp., Armonk, NY, USA).

### Ethical considerations

All study protocols were approved by the institutional review board of Okayama University Graduate School of Medicine, Dentistry and Pharmaceutical Sciences, Japan (approval no. K2111-006). The study was conducted in accordance with the Declaration of Helsinki and institutional data-protection policies.

## Results

### Patient characteristics

Patient characteristics of the 233 patients included in the primary analysis are summarized in Table 1. Median age was 72 years (mean, 70.1 years), and 177 patients (76.0%) were male. The primary tumor sites were the oral cavity in 145 patients (62.2%), hypopharynx in 50 (21.5%), larynx in 19 (8.2%), and p16-negative oropharynx in 19 (8.2%).

**Table 1:** Patient characteristics of the study cohort (n = 233)

Variable		Frequency (n)	Percentage (%)
Sex	Male	177	76.0
	Female	56	24.0
Performance status	0	203	87.1
	≥ 1	30	12.9
Primary site	Oral	145	62.2
	Oropharynx (p16-negative)	19	8.2
	Hypopharynx	50	21.4
	Larynx	19	8.2
Clinical T category	cT1	2	0.9
	cT2	27	11.6
	cT3	76	32.6
	cT4a	128	54.9
Clinical N category	cN0	69	29.6
	cN1	46	19.7
	cN2	118	50.6
Clinical stage	Stage III	54	23.2
	Stage IVA	179	76.8
Pathological T category	pT0–3	126	54.1
	pT4a,b	107	45.9
Pathological N category (n = 223)	pN0,1	125	56.0
	pN ≥ 2	98	44.0
Extranodal extension (n = 223)	Positive	50	22.4
	Negative	173	77.6
Venous invasion	Present	57	24.5
	Absent	176	75.5
Lymphatic invasion	Present	70	42.9
	Absent	163	57.1
Perineural invasion	Present	77	33.0
	Absent	156	67.0
Surgical margin	Positive	48	20.6
	Negative	185	79.4
Neoadjuvant chemotherapy	Yes	56	24.0
	No	177	76.0
Bilateral neck dissection (n = 223)	Yes	89	39.9
	No	134	60.1
Adjuvant therapy	Yes	63	27.0
	No	170	73.0

Pathological nodal status and extranodal extension were assessed in 223 patients who underwent neck dissection

Regarding clinical T classification, cT4a was the most common (126 patients, 54.1%), followed by cT3 (76 patients, 32.6%) and cT2 (27 patients, 11.6%). For clinical N classification, 68 patients had cN0 disease, 46 had cN1, and 118 had cN2 or higher.

Postoperative adjuvant therapy patterns according to pathological risk are summarized in Table 2. Adjuvant therapy was administered in 53 of 77 patients (68.8%) in the high-risk group, including 35 (45.5%) who received concurrent chemoradiotherapy and 18 (23.4%) who received radiotherapy alone. In contrast, among 67 patients classified as intermediate-risk, adjuvant therapy was administered in only 6 patients (9.0%), whereas the majority (91.0%) did not receive postoperative treatment.

**Table 2:** Postoperative adjuvant therapy according to pathological risk

Risk group	CRT	RT	None	Total
High-risk	35	18	24	77
Intermediate-risk	2	4	61	67

High-risk was defined as positive surgical margins and/or extranodal extension. Intermediate-risk was defined as the absence of high-risk features but the presence of venous invasion, lymphatic invasion, or perineural invasion

### Survival outcomes

**Fig. 2:**
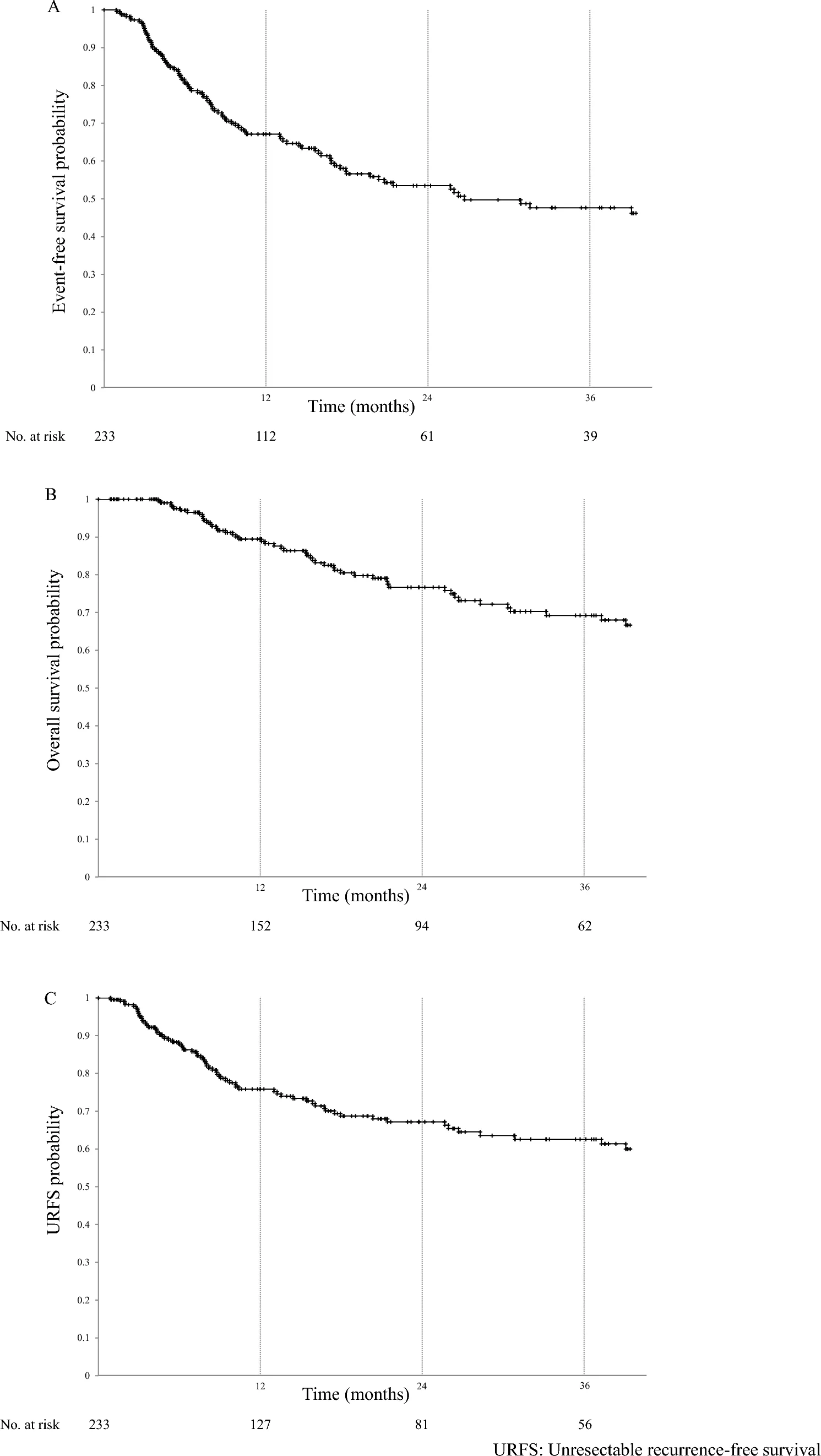
Kaplan–Meier curves for survival outcomes. **A** Event-free survival (EFS), **B** overall survival (OS), and **C** unresectable recurrence-free survival (URFS). The estimated 1-year and 3-year EFS rates were 67.1% and 47.6%, respectively. The corresponding OS rates were 89.4% and 69.2%, and the URFS rates were 75.8% and 62.6%

The median duration of follow-up was 18.2 months (range, 0.9–72.5 months). During follow-up, EFS events occurred in 95 patients, OS events in 49 patients, and URFS events in 69 patients. The corresponding numbers of censored cases were 138, 184, and 164, respectively. One- and 3-year EFS rates were 67.1% and 47.6%, respectively. The corresponding OS rates were 89.4% and 69.2%, respectively, and URFS was analyzed as an exploratory endpoint. The 1- and 3-year URFS rates were 75.8% and 62.6%, respectively (Fig. 2).

### Prognostic factors for EFS

In univariable analyses for EFS, pN2 or higher (*p* < 0.001), extranodal extension (*p* < 0.001), venous invasion (*p* < 0.001), lymphatic invasion (*p* < 0.001), perineural invasion (*p* < 0.001), and positive surgical margins (*p* = 0.024) were all identified as significant adverse prognostic factors (Table 3). In multivariable analysis, pN2 or higher (*p* = 0.023), venous invasion (*p* < 0.001), and perineural invasion (*p* = 0.009) were identified as independent adverse prognostic factors. PS ≥ 1, extranodal extension, and positive margins were significant in univariable analyses, but were not retained as independent factors after multivariable analysis. Further, a sensitivity analysis using lymphatic invasion instead of venous invasion yielded similar results, confirming the robustness of the findings (Table 4, Table S1).

**Table 3:** Univariable Cox proportional hazards analysis for EFS

Variable	HR	95% CI	*p* value
Age (per year increase)	1.01	0.99–1.03	0.259
Sex (Male)	1.32	0.79–2.19	0.288
Performance status (≥ 1)	1.59	0.91–2.78	0.102
Neoadjuvant chemotherapy	1.3	0.84–2.01	0.242
pT4a	1.25	0.82–1.89	0.305
pN ≥ 2	2.56	1.67–3.93	< 0.001
Extranodal extension	2.64	1.66–4.19	< 0.001
Venous invasion	1.76	1.36–2.28	< 0.001
Lymphatic invasion	2.07	1.58–2.70	< 0.001
Perineural invasion	1.6	1.28–2.01	< 0.001
Surgical margin (positive)	1.73	1.08–2.79	0.024
Adjuvant therapy	1.26	0.79–1.99	0.331
*Primary site*			
Oropharynx versus Oral cavity	1.85	0.97–3.53	0.061
Hypopharynx versus Oral cavity	0.89	0.52–1.51	0.661
Larynx versus Oral cavity	0.71	0.31–1.64	0.421

*EFS* Event-free survival

**Table 4:** Multivariable Cox proportional hazards analysis for EFS

Variable	HR	95% CI	*p* value
PS ≥ 1	1.50	0.85–2.66	0.166
pN ≥ 2	1.80	1.09–3.00	0.023
Extranodal extension	1.10	0.60–2.01	0.764
Venous invasion	1.64	1.23–2.19	< 0.001
Perineural invasion	1.38	1.08–1.75	0.009
Surgical margin (positive)	1.27	0.76–2.10	0.359

*EFS* Event-free survival

Multivariable analysis was performed using a Cox proportional hazards model including prespecified clinicopathological variables. Lymphatic invasion and venous invasion were not included simultaneously in the same model to avoid multicollinearity

### Prognostic factors for URFS

In univariable analysis for URFS, PS ≥ 1 (*p* = 0.004), pN2 or higher (*p* < 0.001), extranodal extension (*p* < 0.001), venous invasion (*p* < 0.001), lymphatic invasion (*p* < 0.001), perineural invasion (*p* < 0.001), positive surgical margins (*p* = 0.028), postoperative adjuvant therapy (*p* = 0.012), and p16-negative oropharyngeal cancer (*p* = 0.027) were identified as significant adverse prognostic factors (Table 5). In multivariable analysis, PS ≥ 1 (*p* = 0.043), pathological T4 or higher (*p* = 0.017), pN2 or higher (*p* = 0.010), venous invasion (*p* < 0.001), and perineural invasion (*p* = 0.048) were all identified as independent adverse prognostic factors. Extranodal extension and positive margins were significant in univariable analyses, but were not retained as independent factors in multivariable analysis. Similar trends were observed in a sensitivity analysis using lymphatic invasion instead of venous invasion (Table 6, Table S2).

**Table 5:** Univariable Cox proportional hazards analysis for URFS

Variable	HR	95% CI	*p* value
Age (per year increase)	1.02	1.00–1.04	0.109
Sex (Male)	2.3	1.14–4.65	0.02
Performance status (≥ 1)	2.36	1.31–4.27	0.004
Neoadjuvant chemotherapy	1.29	0.78–2.14	0.322
pT4a	1.65	1.01–2.68	0.045
pN ≥ 2	3.29	1.97–5.48	< 0.001
Extranodal extension	3.84	2.29–6.45	< 0.001
Venous invasion	2.15	1.64–2.83	< 0.001
Lymphatic invasion	2.4	1.78–3.23	< 0.001
Perineural invasion	1.69	1.30–2.19	< 0.001
Surgical margin (positive)	1.84	1.07–3.17	0.028
Adjuvant therapy	1.91	1.15–3.17	0.012
*Primary site*			
Oropharynx versus Oral cavity	2.27	1.10–4.70	0.027
Hypopharynx versus Oral cavity	1.29	0.72–2.32	0.386
Larynx versus Oral cavity	0.75	0.27–2.09	0.577

*URFS* Unresectable recurrence-free survival

**Table 6:** Multivariable Cox proportional hazards analysis for URFS

Variable	HR	95% CI	*p* value
PS ≥ 1	1.92	1.02–3.63	0.043
pT4	1.89	1.12–3.19	0.017
pN ≥ 2	2.15	1.20–3.86	0.01
Extranodal extension	1.28	0.63–2.60	0.493
Venous invasion	2.24	1.53–3.29	< 0.001
Perineural invasion	1.34	1.00–1.80	0.048
Surgical margin (positive)	1.09	0.61–1.95	0.764

*URFS* Unresectable recurrence-free survival

Multivariable analysis was performed using a Cox proportional hazards model including prespecified clinicopathological variables. Lymphatic invasion and venous invasion were not included simultaneously in the same model to avoid multicollinearity

### Patterns of recurrence and salvage treatment

Recurrence was identified in 81 patients (34.8%), comprising local recurrence in 30 patients, regional in 22, distant in 23, and combined in 6. Among these 81 patients with recurrence, disease control at the last follow-up was achieved in 50 patients (61.7%) following curative-intent salvage treatment. Control rates according to recurrence site were 63.3% (19/30) for local recurrence, 77.3% (17/22) for regional recurrence, 56.5% (13/23) for distant metastasis, and 16.7% (1/6) for combined recurrence, with no statistically significant differences among the groups (*p* = 0.053).

**Fig. 3:**
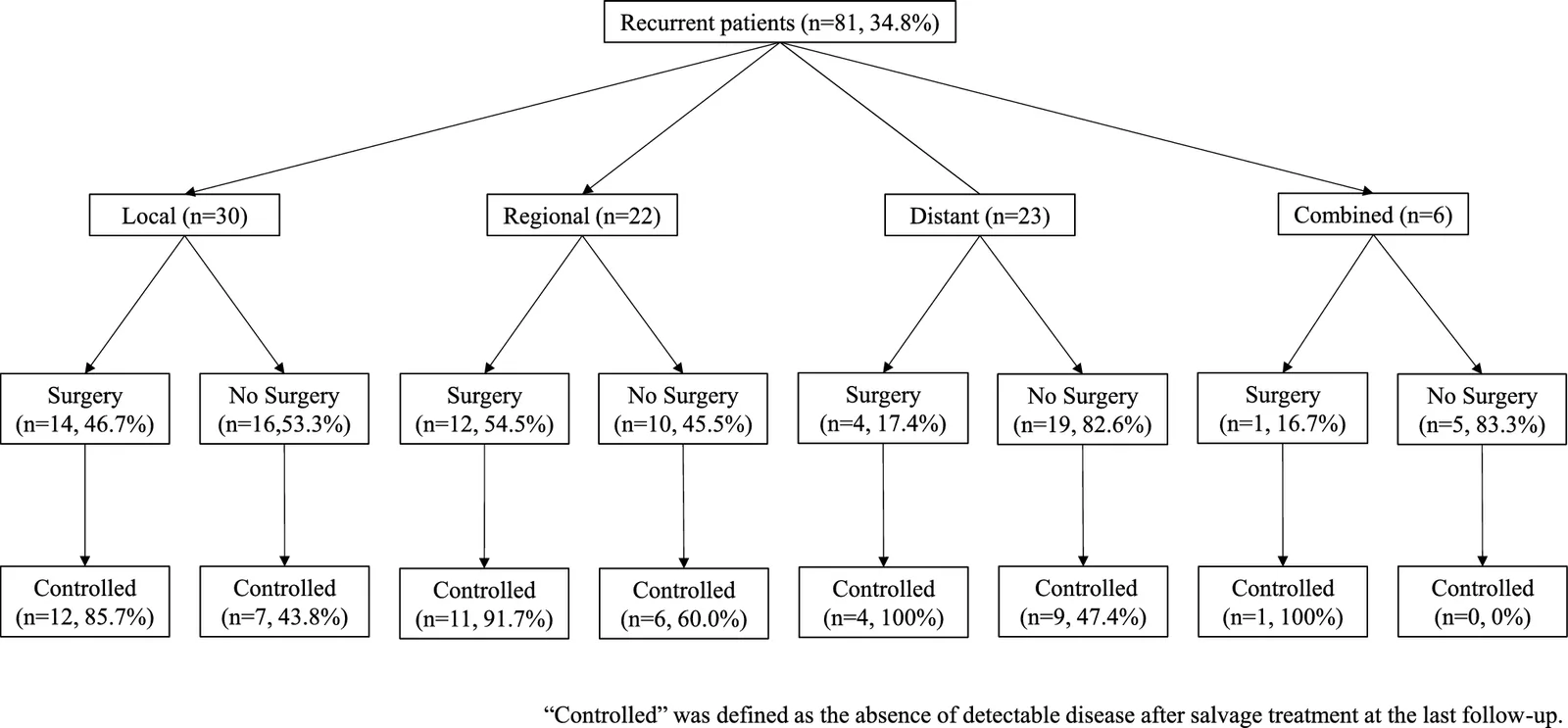
Recurrence patterns and outcomes according to treatment strategy. Among 233 patients, 81 (34.8%) developed recurrence, including local (n = 30), regional (n = 22), distant (n = 23), and combined recurrence (n = 6). Among patients with local recurrence (n = 30), salvage surgery was performed in 14 (46.7%) patients and not performed in 16 (53.3%). Of those who underwent salvage surgery, disease control at the last follow-up was achieved in 12 (85.7%) patients, whereas among those who did not undergo surgery, disease control at the last follow-up was achieved in 7 (43.8%) patients. Among patients with regional recurrence (n = 22), salvage surgery was performed in 12 (54.5%) patients and not performed in 10 (45.5%). Disease control at the last follow-up was achieved in 11 (91.7%) patients who underwent salvage surgery and in 6 (60.0%) patients who did not. Among patients with distant recurrence (n = 23), salvage surgery was performed in 4 (17.4%) patients and not performed in 19 (82.6%). Disease control at the last follow-up was achieved in all 4 patients who underwent salvage surgery and in 9 (47.4%) patients who did not. Among patients with combined recurrence (n = 6), salvage surgery was performed in 1 (16.7%) patient and not performed in 5 (83.3%). Disease control at the last follow-up was achieved in the patient who underwent salvage surgery and in none of the patients who did not “Controlled” indicates absence of detectable disease after curative-intent salvage treatment at last follow-up. These data are presented descriptively and do not imply a causal relationship between treatment selection and outcomes

Salvage surgery was performed in 31 patients (local, n = 14; regional, n = 12; distant, n = 4; combined, n = 1). Among these, disease control at the last follow-up was achieved in 28 patients (90.3%). Control rates following salvage surgery were 85.7% (12/14) for local recurrence, 91.7% (11/12) for regional recurrence, 100% (4/4) for distant metastasis, and 100% (1/1) for combined recurrence (Fig. 3). Re-recurrence after salvage surgery was observed in 1 patient during follow-up. The median follow-up duration after salvage surgery was 21.5 months (range, 1–65 months).

**Fig. 4:**
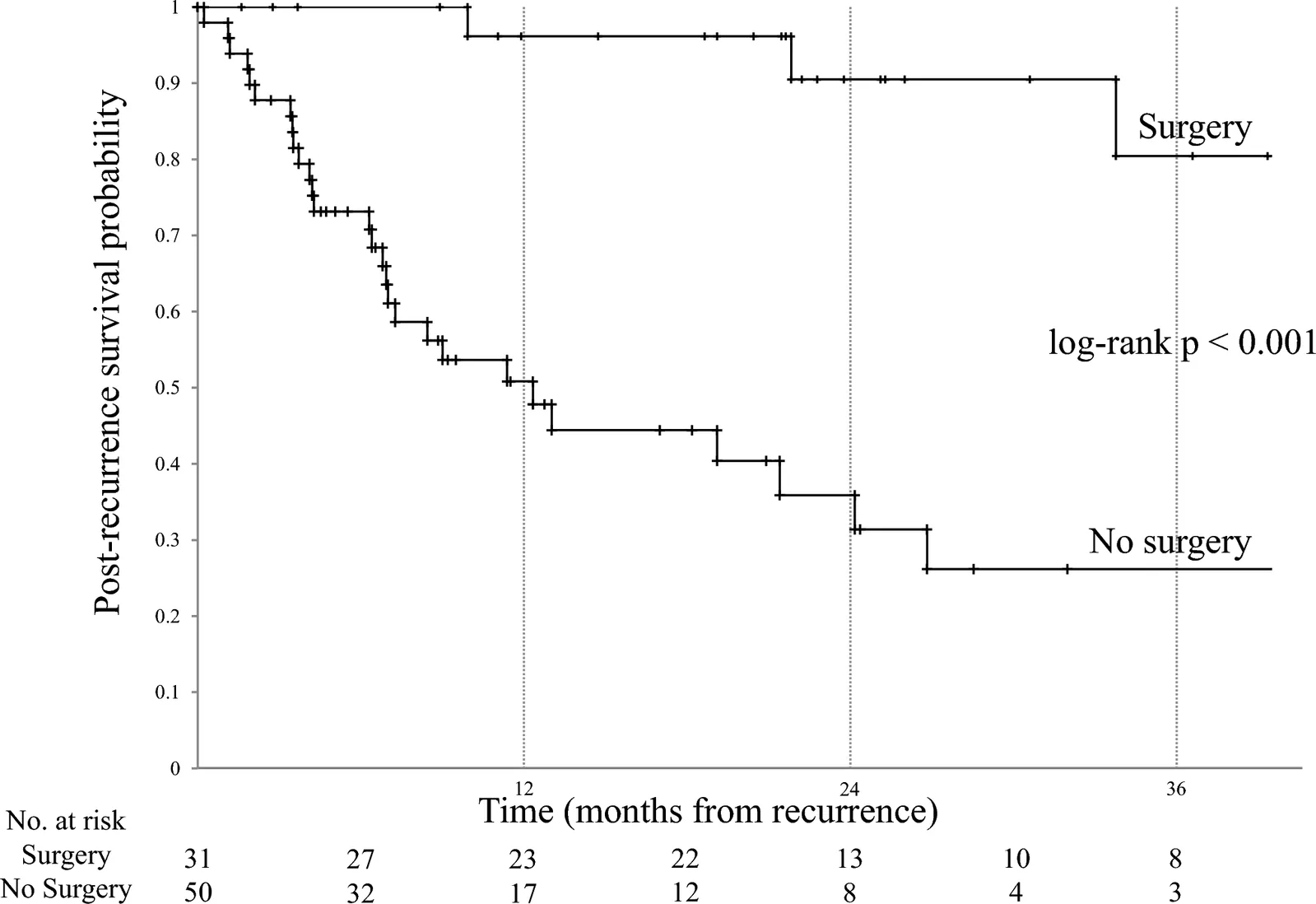
Kaplan–Meier curves for post-recurrence survival (PRS) according to salvage surgery Post-recurrence survival (PRS) was significantly better in patients who underwent salvage surgery than in those who did not (log-rank test, *p* < 0.001)

In the PRS analysis comparing patients who underwent salvage surgery with those who did not, the salvage surgery group demonstrated significantly better survival outcomes (log-rank test, *p* < 0.001) (Fig. 4).

## Discussion

The present study evaluated surgical outcomes in patients with resectable locally advanced HNSCC prior to the introduction of perioperative immunotherapy, using EFS and OS as primary endpoints. This provided real-world surgical outcome data for interpreting outcomes in the era of perioperative immunotherapy. In addition, URFS was introduced as an exploratory metric to describe the heterogeneity of post-recurrence clinical courses, particularly in terms of salvageability, rather than as a surrogate survival endpoint.

In this cohort, the 3-year OS rate was 69.2%, which was relatively favorable compared with previous reports [17, 18]. Conversely, the 3-year EFS rate was 47.6%, indicating that recurrence remains a major clinical challenge. The discrepancy between OS and EFS suggests that, although surgery-based multimodal treatment can achieve a certain level of long-term survival, both prevention of recurrence and management after recurrence remain critical determinants of outcome. Even among patients undergoing salvage treatment, reported 5-year survival rates remain limited (10–40%) [19, 20]. In this context, the EFS benefit observed with perioperative pembrolizumab in the KEYNOTE-689 trial is of particular clinical relevance [11].

In the present study, disease control at the last follow-up was achieved in 61.7% of recurrent cases following curative-intent salvage treatment, and salvage surgery was associated with significantly improved PRS. Although this association is subject to selection bias, as patients selected for salvage surgery tend to display more favorable clinical characteristics, these findings suggest that appropriate post-recurrence interventions may influence post-recurrence clinical course in selected patients.

In the analysis of prognostic factors, pN2 or higher, venous invasion, and perineural invasion were identified as factors independently associated with adverse prognosis in terms of both EFS and URFS. These factors are well-established markers of tumor aggressiveness and metastatic potential [21–24]. Lymphatic and venous invasion reflect tumor dissemination via lymphatic and hematogenous routes, respectively, and may indicate the presence of micrometastatic disease that is currently undetectable using conventional imaging techniques [25, 26]. The association of these features with both recurrence and unresectable recurrence suggests that patients with high-risk pathological characteristics may harbor disease that is difficult to control using surgery alone. Notably, venous invasion may be biologically relevant to the disease processes targeted by perioperative immunotherapy and may overlap with the population included in the KEYNOTE-689 trial, supporting their relevance as adverse pathological features in the perioperative setting.

Extranodal extension was significant in univariable analysis but not retained in the multivariable model after adjusting for nodal burden, suggesting overlapping biological information. In addition, treatment-related confounding may also have contributed, as patients with extranodal extension were more likely to receive postoperative chemoradiotherapy. Venous and lymphatic invasion were not included simultaneously due to multicollinearity. However, sensitivity analyses yielded consistent results, supporting the robustness of the findings. In addition, PS ≥ 1 and pathological T4 or higher were associated with worse URFS, indicating that both tumor burden and host factors influence post-recurrence treatment feasibility. The association between postoperative adjuvant therapy and worse URFS in univariable analysis likely reflects bias in treatment selection rather than any true adverse effect. The observed association with sex should also be interpreted with caution due to limited biological plausibility.

Regarding recurrence patterns, 81 patients (34.8%) developed recurrence, and salvage surgery was performed in 31 (38.3%). Patients who underwent salvage surgery demonstrated significantly better PRS than those who did not. Previous studies have similarly reported associations between salvage surgery and improved survival in patients with recurrent head and neck cancer [27]. This finding was undoubtedly influenced by indication and selection biases, as selection for salvage surgery was based on factors including recurrence site, disease extent, metastatic burden, and patient general condition. Nevertheless, these findings support the existence of a subset of patients with recurrent disease who remain amenable to curative-intent intervention. Recurrence patterns differed by site, with surgical intervention more frequently applied in local and regional recurrence and less commonly in distant metastasis. Distant recurrence generally carries a poor prognosis and is often not amenable to surgery [28]. However, in selected cases with limited metastatic burden, favorable outcomes have been reported [29], consistent with our findings. This finding must be interpreted with caution due to the small number of patients undergoing salvage surgery for distant metastases. These results highlight the heterogeneity of recurrent HNSCC and the presence of a surgically treatable subset. These data may serve as a reference for evaluating how perioperative immunotherapy could alter recurrence patterns, resectability, and subsequent treatment strategies.

An important limitation of this study is the relatively low use of postoperative adjuvant therapy, particularly in patients with intermediate-risk pathological features. At our institution, adjuvant therapy is routinely recommended for high-risk disease, whereas postoperative radiotherapy was generally not administered for intermediate-risk disease at our institution during the study period, reflecting an institutional treatment strategy favoring surgery-based management. As a result, the proportion of patients receiving postoperative radiotherapy in this cohort may be lower than that reported in clinical trials or other institutional series. Furthermore, a subset of patients with high-risk features did not receive postoperative adjuvant therapy, predominantly due to clinical considerations such as advanced age, comorbidities, or patient preference, indicating the presence of treatment selection bias. This variability in postoperative treatment may have influenced both event-free survival and recurrence patterns. In particular, the relatively low use of adjuvant radiotherapy may have contributed to a higher incidence of locoregional recurrence, which may also have affected the observed salvageability patterns. Therefore, the observed salvageability rates should be interpreted with caution, as they may partly reflect institutional treatment patterns rather than intrinsic tumor biology alone.

Several limitations of this study should be acknowledged. First, this was a retrospective study of patients from a single institution, and the possibility of selection biases cannot be excluded. Second, inclusion of recent cases may have limited follow-up duration, particularly for the estimation of longer-term survival outcomes. Therefore, the reported 3-year survival estimates should be interpreted cautiously. Third, because this study used the date of surgery as the starting point and did not include the preoperative period, EFS did not account for progression preventing surgery. Therefore, although conceptually similar, the EFS definition differs from that used in the KEYNOTE-689 trial, and direct comparisons should be interpreted with care. This difference may lead to a relative overestimation of EFS compared with trial-defined EFS. In addition, differences in patient populations, treatment protocols, and assessment schedules may have influenced the results. Fourth, preoperative and post-recurrence treatments were not standardized. Caution is warranted when comparing the present findings with those of perioperative immunotherapy trials, such as KEYNOTE-689, in which postoperative radiotherapy was protocol-mandated. Fifth, the assessment of unresectable recurrence included clinical judgment and incorporated both tumor- and patient-related factors, which may have limited reproducibility. Therefore, URFS should not be interpreted as a surrogate survival endpoint. In addition, URFS did not account for functional outcomes, quality of life, or treatment-related morbidity, which may also substantially influence patient-centered outcomes after recurrence. Therefore, both overall survival and salvage-related outcomes should be interpreted cautiously, particularly with regard to long-term durability. Accordingly, further validation of URFS in independent external cohorts is warranted.

## Conclusion

This study provides real-world surgical outcomes for resectable locally advanced head and neck squamous cell carcinoma prior to the introduction of perioperative immunotherapy, evaluated using EFS and OS. Recurrence remains a major clinical challenge, particularly among patients with high-risk pathological features, with substantial heterogeneity in post-recurrence outcomes according to treatment feasibility. These findings may provide institutional reference data for evaluating treatment outcomes in the era of perioperative ICIs.

## Supplementary Information

Below is the link to the electronic supplementary material.

Supplementary file1 (XLSX 10 KB)

Supplementary file2 (XLSX 10 KB)

## Data Availability

The data that support the findings of this study are available from the corresponding author upon reasonable request, subject to institutional and ethical restrictions due to patient privacy.
